# Acute Necrotizing Encephalopathy of Childhood (ANEC): A Case Report

**Published:** 2017

**Authors:** Afagh HASSANZADEH RAD, Vahid AMINZADEH

**Affiliations:** 1 Pediatrics Growth Disorders Research Center, School of Medicine, 17th Shahrivar Hospital, Guilan University of Medical Sciences, Rasht, Iran

**Keywords:** Anec, Encephalopathy, Thalamus, Brain Stem

## Abstract

Acute Necrotizing Encephalopathy of childhood (ANEC) is a specific type of encephalopathy. After viral infection, it can be diagnosed by bilateral symmetrical lesions predominantly observed in thalami & brainstem of infants & children. Although, it is commonly occurred in Japanese and Taiwanese population. The goal of this article is to report a rare case of ANEC in a 15 months old girl infant from Thaleghani Hospital, Ramian, Gorgan, northern Iran.

## Introduction

Acute Necrotizing Encephalopathy of childhood (ANEC) is a specific type of encephalopathy reported initially by Mizughuchi from Japan in 1995 (1). It is commonly occurred in Japanese and Taiwanese population. It can be diagnosed by bilateral symmetrical lesion in thalami, brain stem, periventricular white matter, and cerebellum. It is most commonly located in brainstem & thalami (2) and occurs following viral infections. Neuropathologically, viral infections cause focal breakdown in blood brain barrier, which mentioned as Dysoria. Besides, ANEC can cause nonspecific changes in liver. 

Acute encephalopathy following viral infection with seizure & altered of consciousness, and absence of CSF pleocytosios with occasional increased level of protein are clinical characteristics of ANEC. 

Bilateral symmrtical lesion in thalami, brainstem, periventricular white matter, putamen & cerebellum can be seen in brain MRI. Mild increase in the level of hepatic aminotransfrase with normal blood amonia can be noticed as its laboratory findings. 

Clinicians should rule out other causes of encephalopaties including Reye syndrome, hemorrhagic shock encephalopathy syndrome, Leigh encephalopathy, organic academia, hypoxia, acute dessiminated encephalomyelitis, venus & arterial infarction in patients (3). 

The goal of this article is to report a rare case of ANEC in north part of Iran.

## Case report

A 15 months old girl infant from referred to Thaleghani Hospital, Ramian, Gorgan, northern Iran, by decreased level of consciousness & repeated convulsion & loss of motor & cognition. The patient had history of upper respiratory infection. Past medical history was unremarkable. Parents were not consanguish & family history of neurological disorders was negative. She weighed 12 kg and her head circumference was 48 cm. In mental status exam, she had no good response to environment, was irritable and had continuously upward gazing. Cranial nerve exam was normal. Deep tendon reflexes increased in upper and lower limbs & she had bilateral Babinsky sign. There was no focal neurological finding. In addition, CBC, metabolic screening and electrolyte were normal. Liver function tests were noted as mild increase in aminotransferase level.

In CSF exam, WBC:2, RBC:2, Glucose:80mg/dl, protein:40mg/dl were noted and all cultures were negative. According to the mentioned CSF analysis and culture and normal PCR for herpes, the encephalitis was ruled out.

**Fig 1:**
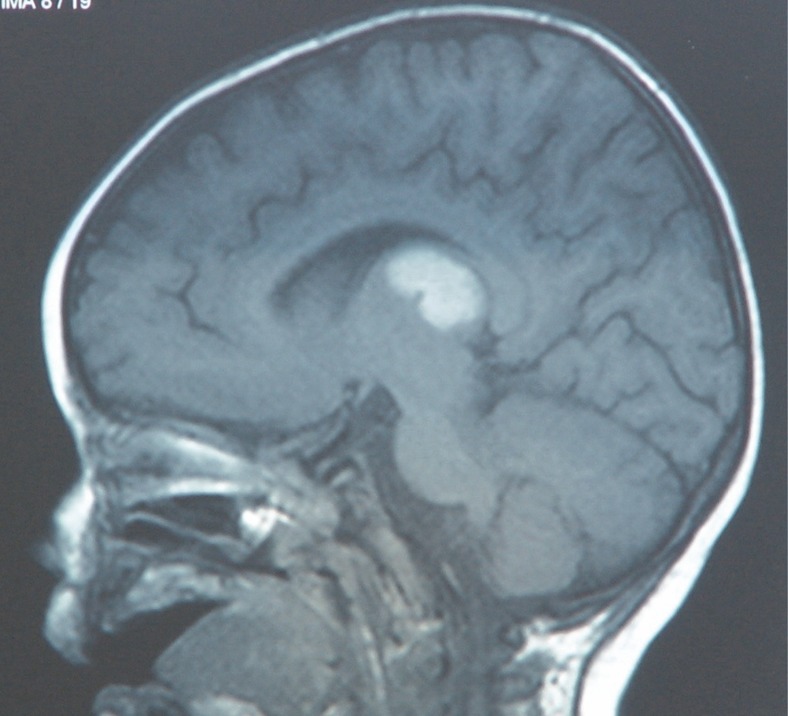
The sagittal view of brain MRI with hyper signal intensity lesion in both thalamus

**Fig 2:**
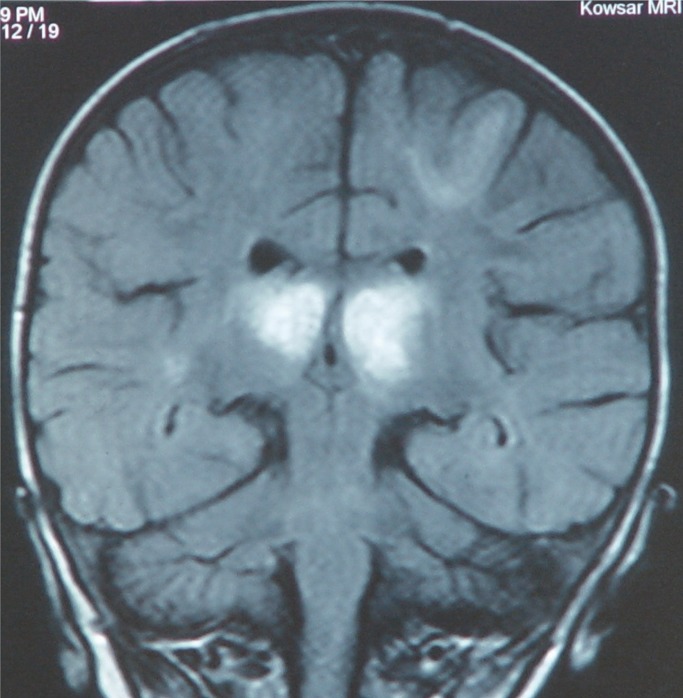
The coronal view of brain MRI with bilateral signal intensity in both thalamus

 In EEG, she had relative increase of slow wave activity. MRI showed increased signal in both thalami symmetrically (Figure 1& 2). Clinician started 20 mg/ kg/d methylprednisolon for 3 d and continued by 400 mg/kg/d IVIG for 5 d as well as anticonvulsive drug with phenobarbital. Clinical condition became better, convulsion was stopped & motormilestone & cognition slowly progressed. After 3 months, she had good motor & cognitive ability.

## Discussion

ANEC is a rare brain disorder that occurs after viral infection reported predominantly from Far East especially from Japan &Taiwan. During 10 yr, 14 cases of ANEC were reported in Korean population (4). 

Main features of the encephalopathy are previous normal development. The presented case had normal psychomotor and regression after viral infection. Differential diagnoses were encephalitis & meningitis but the case had normal CSF analysis and culture and normal PCR for herpes. Sometimes encephalitis can involve some area of brain symmetrically (7). 

Japanese encephalitis can involve thalami bilatrally but the disease is endemic and is not symmetrical (as in ANEC) and other areas such as hypocamp, basal ganglia, substantia nigra, cerebellum and white matter also involved in Japanese encephalitis (8, 9). 

Encephalopathy syndrome in hemorrhagic shock has high fever, kidney dysfunction and shock. EEG shows electrical storm (10). In Reye syndrome, history of aspirin consumption after viral infection especially influenza with liver &kidney dysfunction can be noted. In this case, there is normal (Arterial Blood Gas) ABG so organic academia is ruled out. With normal lactate, mitochondrial disease is also ruled out. In ADEM, there is no symmetrical lesion but patchy demyelization in white matter grey matter and junction is seen.

**In conclusion,** acute encephalopathy in a child with normal previous psychomotor milestone with normal metabolic test & CSF exam and typical neurologic findings was due to ANEC in this report.
